# Biosynthesis of Copper Oxide and Silver Nanoparticles by Bacillus Spores and Evaluation of the Feasibility of Their Use in Antimicrobial Paints

**DOI:** 10.3390/ma16134670

**Published:** 2023-06-28

**Authors:** Arkan Alali, Afrouzossadat Hosseini-Abari, Abbas Bahrami, Maryam Yazdan Mehr

**Affiliations:** 1 Department of Cell and Molecular Biology & Microbiology, Faculty of Biological Science and Technology, University of Isfahan, Isfahan 81746-73441, Iran; 2 Department of Materials Engineering, Isfahan University of Technology, Isfahan 84156-83111, Iran; a.n.bahrami@iut.ac.ir; 3 Faculty EEMCS, Delft University of Technology, Mekelweg 4, 2628 CD Delft, The Netherlands

**Keywords:** silver nanoparticles, copper oxide (CuO) nanoparticles, biosynthesis, NP-modified paints

## Abstract

Modification of paint with nanoparticles (NPs) provides self-cleaning, water/dirt-repellent, and other properties. Therefore, the aim of the present study was to biosynthesize silver (Ag) and copper oxide (CuO) NPs and to prepare NP-modified paint. To this end, AgNPs and CuONPs were biosynthesized using *Bacillus atrophaeus* spores and commercial and crude dipicolinic acid (DPA) extracted from the spore of this bacterium. The synthesized NPs were characterized using electron microscopy, Fourier-transform infrared (FTIR), X-ray diffraction analysis (XRD), and energy-dispersive X-ray spectroscopy (EDS) methods. A minimum inhibitory concentration (MIC) assay of NPs against *Escherichia coli* ATCC8739 and *Staphylococcus aureus* ATCC6538 was carried out. The antibacterial effects of prepared NP–paint complexes were assessed using an optical density (OD) comparison before and after adding metal sheets coated with NP–paint complexes into the nutrient broth medium. Four different types of NPs were synthesized in this research: AgNPs synthesized by spore (A), AgNPs synthesized by commercial DPA (B), AgNPs synthesized by crude DPA (C), and CuONPs synthesized by spore (D). SEM analysis confirmed the spherical shape of NPs. According to the results, NPs A, B, and D showed higher antibacterial activity against *S. aureus* compared to *E. coli*. Furthermore, the analysis of the antibacterial effects of NP–paint complexes suggested that paint–NPs A, B, and C displayed higher activity on *E. coli* compared to *S. aureus*. Moreover, the antibacterial effect of paint–NP D was significantly lower than other NPs. According to this robust antibacterial effect on pathogenic bacteria, it seems that these NP–paint complexes could be useful in public places such as hospitals, airports, dormitories, schools, and office buildings, where the rate of transmission of infection is high.

## 1. Introduction

Currently, nanoparticles (NPs) play a critical role in different aspects of human life, such as medicine, agriculture, food, and various other industries. The quality of nanoparticles is highly affected by their synthesis method. Several methods have been introduced to improve the properties of NPs, which can be categorized into three main classes: chemical, physical, and biological methods [1]. A major concern in the chemical/physical synthesis of NPs is the use of chemical additives and compounds, including but not limited to agents such as 2-mercaptoethanol, 1-thioglycerol, thioglycolic acid (TGA), and thiolactic acid. Most of these compounds are toxic to the environment and humans. In addition to the harmful impacts on the environment, high energy consumption, low productivity, and larger particles are other downsides of physicochemical synthesis methods [2]. For these reasons, green synthesis of NPs has emerged as a new alternative approach in this particular area. Various types of biological materials including plant extracts [3], agricultural wastes [4], fungi, bacteria, and algae have been used successfully to synthesize different NPs [5]. In most biological methods, bacterial cells play a central role in the green synthesis of NPs due to their diversity and their ability to adapt themselves to harsh environmental conditions [6]. More importantly, individual bacteria are capable of converting toxic metal ions into nontoxic components, using reductase enzymes and reducing equivalents such as NADH and NADPH [7]. The biosynthesis of NPs by bacteria takes place via two different pathways: intercellular and extracellular. Extracellular synthesis of NPs is more controlled by culture conditions such as temperature, shaking rate, pH, and substrate composition/concentration. In addition, the extracellular biosynthesis of NPs is more favorable because the extraction of NPs is easier in this condition; more importantly, synthesized NPs are more resistant to oxidation [8]. *Lactobacillus plantarum* [9], *Aeromonas hydrophila* [10], *Bacillus subtillis* [11], *Bacillus endophyticus* [12], and *Deinococcus radiodurans* [13] are examples of bacterial species that have been successfully used for the synthesis of various types of NPs such as ZnO, iron oxide, and silver. Silver (Ag) nanoparticles (AgNPs) are among the best-known metallic NPs, when it comes to antibacterial applications. The synthesis of AgNPs using physical and chemical methods has been evaluated in many studies. Furthermore, there have been some reports on the green synthesis of AgNPs, using various plants and different bacterial, fungal, and algal species [9,10,11,12,13,14]. Antibacterial, antifungal, antiviral, anticancer, and antioxidant activities of AgNPs have been reported in several studies [15]. Due to these biological activities and their unique optical, electrical, and thermal properties, AgNPs are applied in various fields such as human healthcare, cosmetics, imaging, biosensing, textiles, water treatment, and medicine [15]. Similar biological activities and applications have been suggested for copper oxide nanoparticles (CuONPs) [16].

The preparation of paints with nanoparticle additives is a newly emerging application of nanoparticles in the building industry. It is postulated that the presence of nanoparticles in paints can potentially improve the water/dirt-repellent, anti-graffiti, bactericidal, easy-to-clean, fire-retardant, self-cleaning, scratch-resistant, UV-protective, and thermal insulation properties of paints [17]. Moreover, the idea of antibacterial paints with self-protecting capabilities with potential applications in hospitals, schools, and public areas has attracted substantial attention. The main aim of the current study was the synthesis of a modified antibacterial paint, containing green AgNPs and CuONPs. The synthesis pathway, properties of NPs, and antibacterial properties of the paint are comprehensively discussed.

## 2. Materials and Methods

### 2.1. Biosynthesis of AgNPs and CuONPs Using Bacillus atrophaeus Spores

Firstly, *Bacillus atrophaeus* (*B. atrophaeus*) was cultivated in an MSR medium, containing 2.5% yeast extract, 1.5% Bacto tryptone, 1% glucose, and 0.3% K_2_HPO_4_ and incubated at 37 °C at 200 rpm for 24 h. Then, this preculture was used to inoculate DSM medium containing 0.8% nutrient broth, 0.01 mM MgCl_2_, 0.1% KCl, 0.01 mM FeSO_4_, 1 mM Ca(NO_3_)_2_, and 0.025% MgSO_4_·7H_2_O in 100 mL of distilled water to prepare bacterial spores. Incubation was performed at 37 °C at 200 rpm for 48 h. Finally, the culture broth was centrifuged at 13,000 rpm for 10 min. For the synthesis of AgNPs, the pellet was added into a 250 mL Erlenmeyer flask with 100 mL of distilled water and 0.0017 g of AgNO_3_. The flask was incubated at room temperature for 48 h [18]. The biosynthesis of CuONPs was carried out using CuSO_4_ salt and a similar protocol. The formation of NPs was discerned through a change in the color of the prepared solution. At the end, samples were washed away with acetone to remove biological residues from synthesized powders.

### 2.2. Biosynthesis of AgNPs Using Commercial and Crude Dipicolinic Acid (DPA)

The prepared bacterial spore pellet was added to 1 mL of distilled water and heated using a water bath for 30 min. Then, the solution was centrifuged at 13,000 rpm for 2 min, and the supernatant was used as crude DPA. For the synthesis of silver NPs, 1 mL of crude DPA was added into a 250 mL Erlenmeyer flask containing 100 mL of distilled water and 0.0017 g of AgNO_3_. The flask was incubated at room temperature for 1 h. Biosynthesis of AgNPs using commercial DPA (2,6-pyridinedicarboxylic acid, P63808 Sigma, Hamburg, Germany) was conducted using the same method. Briefly, 0.017 g of standard DPA was added to 100 mL of distilled water containing 0.0017 g of AgNO_3_ and incubated [18]. At the end, samples were washed away with acetone to remove biological residues from synthesized powders.

### 2.3. Characterization of Synthesized NPs

UV/Vis spectroscopy in the range of 200–600 nm was used to confirm the formation of NPs. The size and morphology of synthesized NPs were evaluated using a scanning electron microscope (SEM) (XI30 Philips, Eindhoven, The Netherlands). For this aim, the AgNPs were centrifuged at 10,000 rpm for 30 min, and the sediment was dissolved in ethanol and washed with distilled water to obtain the pellet. Finally, the pellet was dried, and thin films of it were prepared on carbon-coated copper grids [19]. The grid was used to fix particles on a substrate.

Functional groups present in AgNPs were analyzed using Fourier-transform infrared (FTIR) method. A thin film of as-synthesized AgNPs was prepared by mixing NPs with KBr powder (1:4) and analyzed using an FT-IR spectrophotometer (8400 S, Shimadzu, Kyoto, Japan) at 400–4000 cm^−1^. The crystallographic structure of NPs was determined using XRD analysis (Philips X-Pert Pro diffractometer, Philips, Eindhoven, The Netherlands). To this end, nanoparticles were centrifuged at 10,000 rpm for 15 min, and the pellet was washed using distilled water. Then, the pellet was dried, and XRD analysis was conducted using CuKα radiation in a scan range of 10 < 2θ < 80. The elemental constituents of the AgNPs were examined using energy-dispersive X-ray (EDS) analysis. To this aim, nanoparticles were centrifuged at 10,000 rpm for 15 min, and the pellet was washed using distilled water and analyzed using SEM (XL 30; Philips, Eindhoven, The Netherlands). SEM imaging (Philips, Eindhoven, The Netherlands) was carried out within the voltage range of 10 to 25 kV with working distance around 10 mm. Particles were also characterized using a transmission electron microscope (TEM, JEOL JSM-6710F, Tokyo, Japan).

### 2.4. Determination of Minimum Inhibitory Concentration (MIC) of NPs

The serial dilution method using 96-well plates was employed to determine the minimum inhibitory concentrations (MICs) of biosynthesized NPs against *Staphylococcus aureus* (*S. aureus*) ATCC 6538 and *Escherichia coli* (*E. coli*) ATCC 8739. Serial twofold dilutions of NPs in concentrations ranging from 1000 μg to 31.25 μg were used to determine MIC. Firstly, 100 μL of nutrient broth medium was added into wells. Then, 100 μL of NPs at a concentration of 1000 μg/mL was added into the first well, and serial dilution was performed. Finally, 100 μL of bacterial suspension (0.5 McFarland) was added into the wells. The microplates were incubated at 37 °C for 24 h, and the change in resazurin color from blue to pink was used as a growth detector. The lowest concentration without visible growth was defined as the MIC. To prepare the resazurin solution, 0.002 g of this compound was added to 10 mL of distilled water and then sterilized using a 0.22 μm membrane filter [20].

### 2.5. Preparation of Paint–NP Complexes

**Figure 1:**
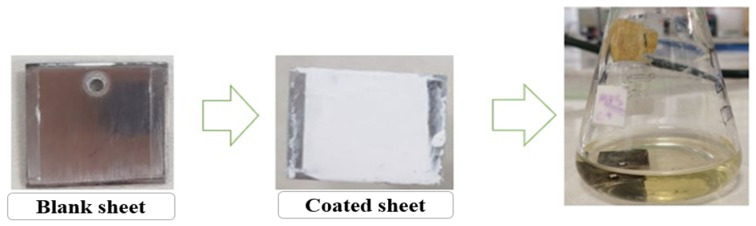
Preparation of metal sheets coated with paint–NP complexes for the antibacterial test.

Initially, the NPs were added to 100 μL of distilled water and sonicated using an ultrasonic bath for 5 min to disperse NPs. Then, 0.5 mL of commercial paint was blended with NP solution, and the obtained complexes were brushed onto the sterilized metal sheets. Steel sheets (2 cm × 2 cm) were used for this aim. The prepared complexes were sterilized under UV light for 30 min and transformed into nutrient broth medium for antibacterial analysis (Figure 1).

### 2.6. Examination of Antibacterial Effects of Prepared Paint–NP Complexes

For this aim, metal sheets coated with NP-containing paints were added into 100 mL Erlenmeyer flasks containing 20 mL of nutrient broth medium. Flasks were incubated at 37 °C for 8 h, and the optical density (at 600 nm) of their content was recorded every 2 h. Then, 100 μL of culture broth was transferred onto the nutrient agar plates; after incubation at 37 °C for 24 h, the number of colonies was determined.

## 3. Results and Discussion

### 3.1. Biosynthesis and Characterization of AgNPs and CuONPs

In this research, four different types of NPs were synthesized: AgNPs synthesized by spore (A), AgNPs synthesized by commercial DPA (B), AgNPs synthesized by crude DPA (C), and CuONPs synthesized by spore (D). Table 1 summarizes the sample information.

**Figure 2:**
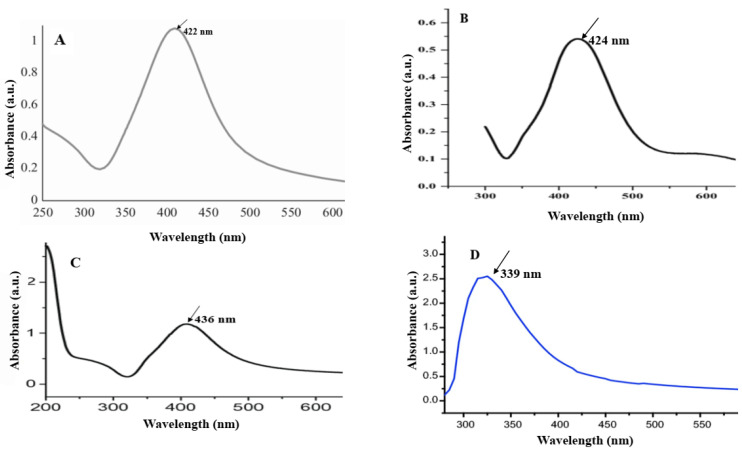
UV/Vis spectra of AgNPs A (**A**), B (**B**), and C (**C**), and CuONPs (**D**).

Firstly, the formation of NPs was confirmed using the UV/Vis technique. Metal NPs have their characteristic surface plasmon resonance (SPR) peaks. For example, the SPR peak of silver NPs appears between 400 and 450 nm. This value for CuONPs is 250–350 nm. As shown in Figure 2, the SPR peaks of AgNPs A, B, and C appeared at 422, 424, and 436 nm, respectively. This variation in peak position is related to the size and shape of NPs. Furthermore, a peak at 339 nm was observed in the UV/Vis spectrum of CuONPs, which confirmed the presence of CuONPs [21,22].

**Figure 3:**
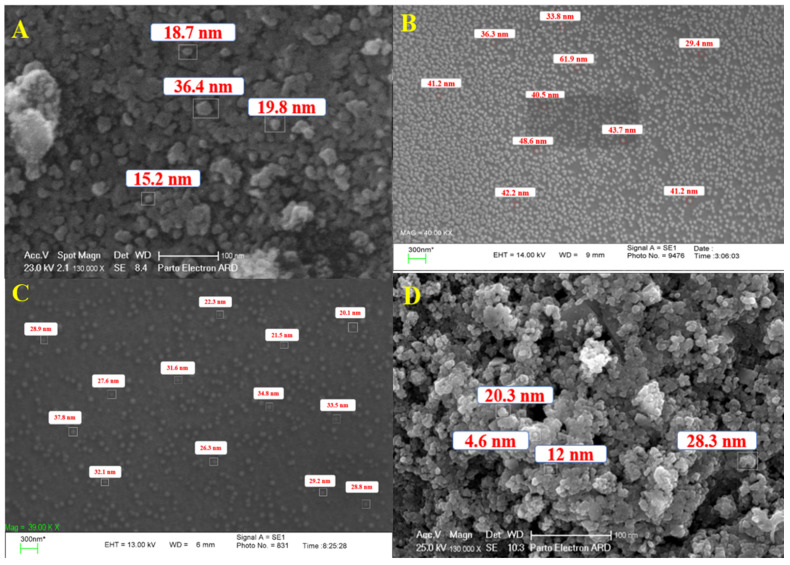
Scanning electron microscopy graphs of silver NPs A (**A**), B (**B**), and C (**C**), and copper oxide NPs (**D**).

**Figure 4:**
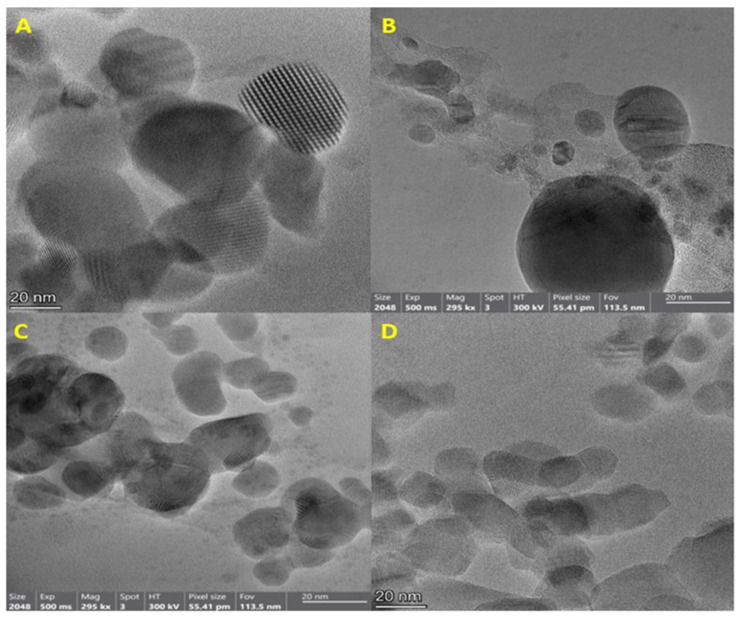
Transmission electron microscopy images of silver NPs A (**A**), B (**B**), and C (**C**), and copper oxide NPs (**D**).

**Figure 5:**
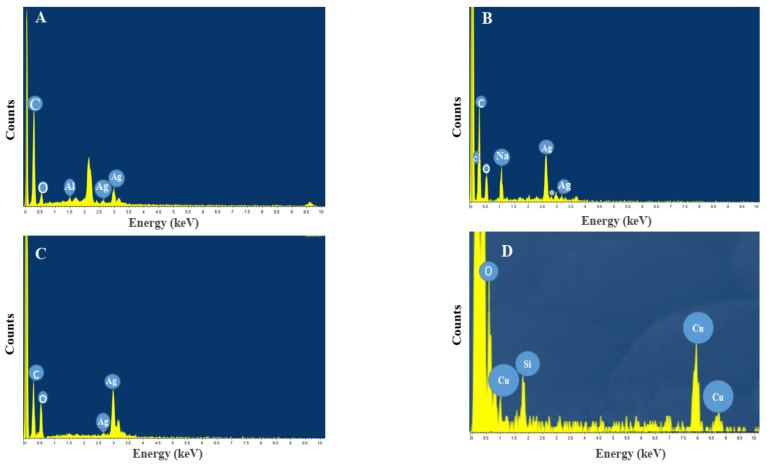
EDS spectra of synthesized silver NPs A (**A**), B (**B**), and C (**C**), and copper oxide NPs (**D**).

Characterization of these NPs using SEM analysis confirmed a rather spherical morphology for NPs. Moreover, the size range of nanoparticles A, B, C, and D was 15–36, 29–61, 20–38, and 4–28 nm, respectively (Figure 3). A comparison of the size range of synthesized AgNPs using DPA showed that nanoparticles synthesized using crude DPA (B) were smaller than nanoparticles synthesized by commercial DPA (C). The size range of synthesized NPs using bacterial spore and its derivatives (in current study) was similar to that of NPs synthesized using other biological methods. For instance, the size range of AgNPs synthesized by *Carica papaya* peel extract, *Aspergillus terreus*, and *Azotobacter vinelandii* was reported to be 28, 1–20, and 20–70 nm, respectively [23,24,25]. In addition, TEM analysis showed that synthesized NPs mainly were of spherical shape and aggregated. AgNPs B were more dispersed (Figure 4). Further analysis of the synthesized NPs was performed using EDS. According to the results, strong signal peaks were observed near 3 keV (Figure 5A–C) which proved the presence of AgNPs [26]. Furthermore, the strong peaks near 1 and 8 keV confirmed the formation of CuONPs.

**Figure 6:**
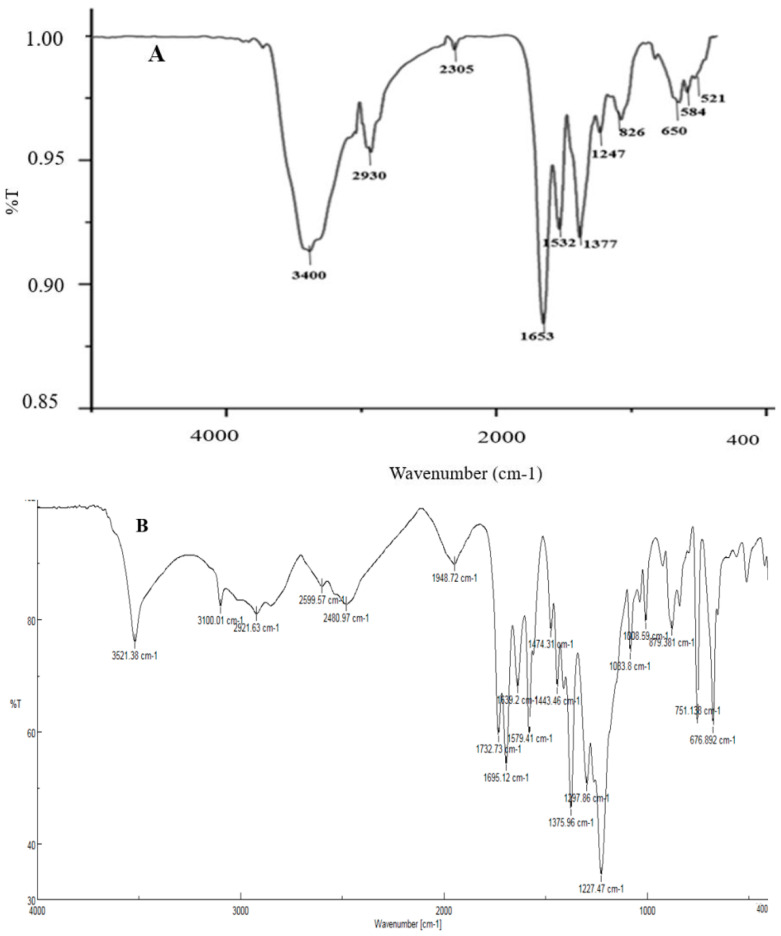

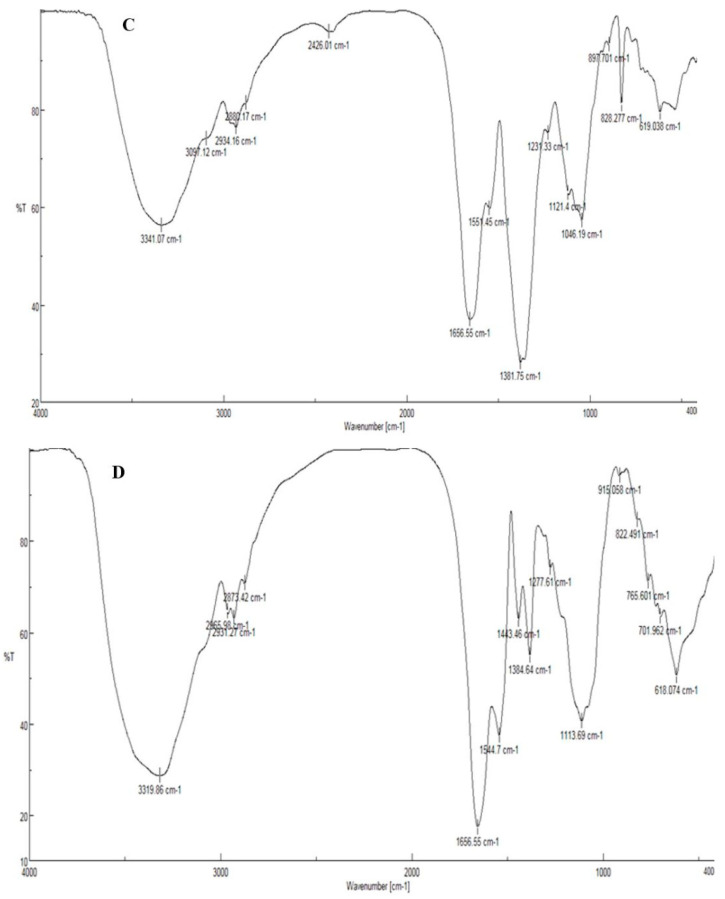
FTIR spectra of synthesized silver NPs A (**A**), B (**B**), and C (**C**), and copper oxide NPs (**D**).

Analysis of functional groups of synthesized NPs was performed using FTIR. FTIR spectrum of AgNPs A showed several peaks at positions 3400, 2930, 1653, 1377, 1247, 826, and 650 cm^−1^ that were attributed to O–H, C–H, O=C=O, N–H, C–H, C–O, C=CH_2_, and –CH=CH groups, respectively. Furthermore, the band at 1532 cm^−1^ was assigned to a combination of N–H deformation and C–N stretching vibrations. The peaks at 584 and 521 cm^−1^ were assigned to AgO (Figure 6A). The FTIR spectrum of AgNPs B showed peaks at 2921 cm^−1^, 1732 cm^−1^, and 1375 cm^−1^, which confirmed the formation of AgNPs (Figure 6B). The FTIR spectrum of AgNPs C showed peaks at 2934 cm^−1^, 1656 cm^−1^, 1381 cm^−1^, and 1121 cm^−1^, which were attributed to C–H stretching, C=O group, C–C (or C–N) stretching, and O–H stretching, respectively. These bonds confirmed the presence of AgNPs (Figure 6C). The FTIR spectrum of CuONPs (Figure 6D) showed several peaks at 1656 cm^−1^, 1384 cm^−1^, 1113 cm^−1^, 765 cm^−1^, and 618 cm^−1^, which were assigned to O–H bending, C–O asymmetric in the structure of CuONPs, C–O symmetric in the structure of CuONPs, and Cu–O stretching vibration, respectively [27,28].

**Figure 7:**
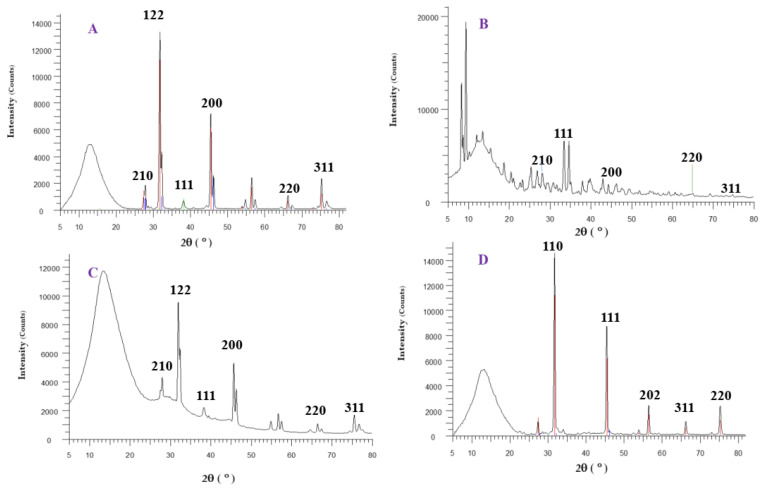
XRD pattern of synthesized silver NPs A (**A**), B (**B**), and C (**C**), and copper oxide NPs (**D**).

The XRD pattern of AgNPs A, B, and C and CuONPs is shown in Figure 7. For AgNPs, several Bragg reflection peaks were observed at 2θ values of 28°, 32°, 38.12°, 46°, 54°, 57°, 64°, and 77° which were indexed to (210), (122), (111), (200), (142), (241), (220), and (311) planes of pure silver based on a face-centered cubic structure [29]. The XRD results clearly show that the AgNPs were successfully synthesized and had a crystalline structure. In addition to AgNP peaks, some other peaks could be ascribed to Ag_2_O. It is important to mention that there was hardly any indication of the Ag nitrate phase (at least not in the detection limit of XRD, which is roughly 5 wt.%), inferring that the synthesis was successfully carried out. In addition, the XRD pattern of CuONPs was observed to be at 2θ = 31.8°, 46°, 57°, 66°, and 75°. These narrow peaks confirmed the crystalline structure of synthesized CuONPs [30].

### 3.2. MIC Determination of NPs

To determine the MIC of as-synthesized NPs against *E. coli* and *S. aureus*, the microdilution method was performed. MIC values are shown in Table 2. On the basis of these results, NPs A, B, and D were more active against *S. aureus* compared to *E. coli*, while the antibacterial effect of NPs C on both was equal. These results are in agreement with previous studies. For example, Erjaee et al. (2017) showed that green-synthesized AgNPs using *Chamaemelum nobile* extract inhibited the growth of pathogenic bacteria, and its MIC against *E. coli* and *S. aureus* was 7.8 μg/mL and 31.2 μg/mL, respectively [31]. In another study, it was shown that AgNPs synthesized using a chemical approach had no antibacterial effects on *S. aureus*, whereas they were highly active against *E. coli* with an MIC of 128 μmol/L [32]. Furthermore, an analysis of the antibacterial activity of CuONPs synthesized using bacterial stains by John et al. showed that these NPs were strongly active against *E. coli* and *S. aureus* with MIC values equal to 25 ± 0.4 and 25 ± 0.2 μg/mL, respectively [33]. The efficacy of the antibacterial activity of AgNPs is due not only to their nanoscale size but also to their large ratio of surface area to volume. They exhibit antibacterial effects through different processes including the production of reactive oxygen species (ROS), cell-wall penetration, and inhibition of DNA replication [34]. The antibacterial activity of CuONPs is related to their capability to produce ROS and to form a complex with peptides inside the target bacteria. The oxidation state plays a key role in this process [35].

### 3.3. Evaluation of Antibacterial Effects of Prepared Paint–NP Complexes

**Figure 8:**
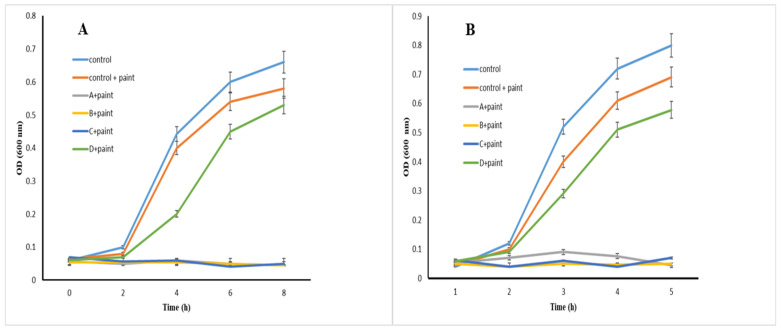
Antibacterial effects of NP–paint complexes on *E. coli* (**A**) and *S. aureus* (**B**).

The antibacterial activity of NP–paint complexes against pathogenic bacterial strains suggested that metal sheets coated with these complexes strongly inhibited the growth of *E. coli* and *S. aureus*. According to Figure 8 and Table 3, both of them were inhibited by paint samples modified with NPs A, B, and C. In addition, the antibacterial activity of these complexes against *E. coli* was higher than *S. aureus*. No growth was observed after treatment of *E. coli* paint–NPs C. In other words, the viability of *E. coli* after treatment with complexes A, B, C, and D was decreased to 1.9%, 1.76%, 0%, and 4.81% compared to the untreated control. Furthermore, complexes A, B, C, and D decreased the viability of *S. aureus* to 8.44%, 5.6%, 3.4%, and 18.5% compared to the control.

Modification of paints with NPs may enhance their physicochemical properties and provides antimicrobial activity, making them applicable in places such as hospital walls (as the source of nosocomial infections), schools, restaurants, office buildings, and laboratories [36]. Hence, this topic has attracted researchers’ attention, and a limited number of studies have been reported in this area in recent years. For instance, Solano et al. (2020) prepared nano-filled paint with titanium oxide (TiO_2_) and zinc oxide (ZnO) NPs and analyzed their physicochemical characteristics and antibacterial activity. They suggested that the modification of paint with TiO_2_ and ZnONPs improved its self-cleaning property of it. In addition, antibacterial analysis against *E. coli* using the disc diffusion method showed that, in the presence of ZnONPs, nano-filled paint presented inhibitory activity, but no antibacterial activity was observed in the presence of TiO_2_NPs. This lower antibacterial activity was attributed to the low capability of NPs to release from paint [37]. This confirmed that the disc diffusion method is not suitable for evaluating the real antibacterial potential of NP–paint complexes. For this reason, here, we used aqueous medium. The antibacterial properties of the synthesized paint were related to Ag/Cu release at the interface. When Ag/Cu ions are released, they can adhere to the negatively charged bacterial cell wall and cause the rupture of the cell structure; this can result in protein denaturation and lead to the demise of the bacteria [38]. Within the bacteria, ions can also bind to DNA structures and influence crosslinking within and between nucleic acid strands, resulting in disruption of the helical structure of the DNA. Furthermore, antibacterial ions are believed to have some negative implications for the critical biochemical processes [39,40,41] needed for the livability of the bacteria [42,43].

## 4. Conclusions

The production of surfaces with antibacterial properties is a research area that has attracted much attention in recent decades. Nanoparticles play a key role in this field. Therefore, we synthesized AgNPs and CuONPs using biological methods to generate paint–NP complexes. Antibacterial analysis using the MIC and colony count methods revealed that these complexes are strongly active against pathogenic bacteria. These results suggest that they could be useful for application in hospitals, hotels, restaurants, and other public places.

## Figures and Tables

**Table 1:** Sample codes and details.

Sample Code	Explanation
A	AgNPs synthesized by spore
B	AgNPs synthesized by commercial DPA
C	AgNPs synthesized by crude DPA
D	CuONPs synthesized by spore

**Table 2:** MIC values of NPs and commercial paint used in the present study.

NPs	MIC (μg/mL)	
	*E. coli*	*S. aureus*
A	125	62.5
B	125	31.2
C	62.5	62.5
D	200	180
Paint	100 mg/mL	100 mg/mL

**Table 3:** Antibacterial activity of NP–paint complexes (after 12 h incubation).

NP–Paint Complexes	Number of Colonies/mL	
	*S. aureus*	*E. coli*
Control	3.73 × 10^5^	3.11 × 10^5^
Control + paint	2.7 × 10^5^	2.13 × 10^5^
A + paint	31.5 × 10^3^	6 × 10^3^
B + paint	21 × 10^3^	5.5 × 10^3^
C + paint	13 × 10^3^	No growth
D + paint	69 × 10^3^	15 × 10^3^

## Data Availability

Not applicable.

## References

[1] Yang R., Hou E., Cheng W., Yan X., Zhang T., Li S., Yao H., Liu J., Guo Y. (2022). Membrane-Targeting Neolignan-Antimicrobial Peptide Mimic Conjugates to Combat Methicillin-Resistant *Staphylococcus aureus* (MRSA) Infections. J. Med. Chem..

[2] Yu H., Zhu J., Qiao R., Zhao N., Zhao M., Kong L. (2022). Facile Preparation and Controllable Absorption of a Composite Based on PMo_12_/Ag Nanoparticles: Photodegradation Activity and Mechanism. Chemistryselect.

[3] Maqbool Q., Czerwinska N., Giosue C., Sabbatini S., Ruello M.L., Tittarelli F. (2022). New waste-derived TiO_2_ nanoparticles as a potential photocatalytic additive for lime based indoor finishings. J. Clean. Prod..

[4] Maqbool Q., Yigit N., Stöger-Pollach M., Ruello M.L., Tittarelli F., Rupprechter G. (2022). *Operando* monitoring of a room temperature nanocomposite methanol sensor. Catal. Sci. Technol..

[5] Gour A., Jain N.K. (2019). Advances in green synthesis of nanoparticles. Artif. Cells Nanomed. Biotechnol..

[6] Wang L., Miao X., Ali J., Lyu T., Pan G. (2018). Quantification of Oxygen Nanobubbles in Particulate Matters and Potential Applications in Remediation of Anaerobic Environment. ACS Omega.

[7] Mittal A.K., Bhaumik J., Kumar S., Banerjee U.C. (2014). Biosynthesis of silver nanoparticles: Elucidation of prospective mechanism and therapeutic potential. J. Colloid Interface Sci..

[8] Gahlawat G., Choudhury A.R. (2019). A review on the biosynthesis of metal and metal salt nanoparticles by microbes. RSC Adv..

[9] Selvarajan E., Mohanasrinivasan V. (2013). Biosynthesis and characterization of ZnO nanoparticles using *Lactobacillus plantarum* VITES07. Mater. Lett..

[10] Jayaseelan C., Rahuman A.A., Roopan S.M., Kirthi A.V., Venkatesan J., Kim S.-K., Iyappan M., Siva C. (2013). Biological approach to synthesize TiO2 nanoparticles using *Aeromonas hydrophila* and its antibacterial activity. Spectrochim. Acta Part A Mol. Biomol. Spectrosc..

[11] Sundaram P.A., Augustine R., Kannan M. (2012). Extracellular biosynthesis of iron oxide nanoparticles by *Bacillus subtilis* strains isolated from rhizosphere soil. Biotechnol. Bioprocess Eng..

[12] Gan L., Zhang S., Zhang Y., He S., Tian Y. (2018). Biosynthesis, characterization and antimicrobial activity of silver nanoparticles by a halotolerant *Bacillus endophyticus* SCU-L. Prep. Biochem. Biotechnol..

[13] Li J., Tian B., Li T., Dai S., Weng Y., Lu J., Xu X., Jin Y., Pang R., Hua Y. (2018). Biosynthesis of Au, Ag and Au–Ag bimetallic nanoparticles using protein extracts of *Deinococcus radiodurans* and evaluation of their cytotoxicity. Int. J. Nanomed..

[14] Beyene H.D., Werkneh A.A., Bezabh H.K., Ambaye T.G. (2017). Synthesis paradigm and applications of silver nanoparticles (AgNPs), a review. Sustain. Mater. Technol..

[15] Zhang X.-F., Liu Z.-G., Shen W., Gurunathan S. (2016). Silver Nanoparticles: Synthesis, Characterization, Properties, Applications, and Therapeutic Approaches. Int. J. Mol. Sci..

[16] Waris A., Din M., Ali A., Ali M., Afridi S., Baset A., Khan A.U. (2021). A comprehensive review of green synthesis of copper oxide nanoparticles and their diverse biomedical applications. Inorg. Chem. Commun..

[17] West G.H., Lippy B.E., Cooper M.R., Marsick D., Burrelli L.G., Griffin K.N., Segrave A.M. (2016). Toward responsible development and effective risk management of nano-enabled products in the U.S. construction industry. J. Nanopart. Res..

[18] Hosseini-Abari A., Emtiazi G., Lee S.-H., Kim B.-G., Kim J.-H. (2014). Biosynthesis of Silver Nanoparticles by *Bacillus stratosphericus* Spores and the Role of Dipicolinic Acid in This Process. Appl. Biochem. Biotechnol..

[19] Banala R.R., Nagati V.B., Karnati P.R. (2015). Green synthesis and characterization of *Carica papaya* leaf extract coated silver nanoparticles through X-ray diffraction, electron microscopy and evaluation of bactericidal properties. Saudi J. Biol. Sci..

[20] Loo Y.Y., Rukayadi Y., Nor-Khaizura M.-A., Kuan C.H., Chieng B.W., Nishibuchi M., Radu S. (2018). In Vitro Antimicrobial Activity of Green Synthesized Silver Nanoparticles Against Selected Gram-negative Foodborne Pathogens. Front. Microbiol..

[21] Anandalakshmi K., Venugobal J., Ramasamy V. (2016). Characterization of silver nanoparticles by green synthesis method using *Pedalium murex* leaf extract and their antibacterial activity. Appl. Nanosci..

[22] Venkataa A.L.K., Anthony S.P., Muthuraman M.S. (2019). Synthesis of *Solanum nigrum* mediated copper oxide nanoparticles and their photocatalytic dye degradation studies. Mater. Res. Express.

[23] Kokila T., Ramesh P., Geetha D. (2016). Biosynthesis of AgNPs using *Carica Papaya* peel extract and evaluation of its antioxidant and antimicrobial activities. Ecotoxicol. Environ. Saf..

[24] Li G., He D., Qian Y., Guan B., Gao S., Cui Y., Yokoyama K., Wang L. (2011). Fungus-Mediated Green Synthesis of Silver Nanoparticles Using *Aspergillus terreus*. Int. J. Mol. Sci..

[25] Karunakaran G., Jagathambal M., Gusev A., Torres J.A.L., Kolesnikov E., Kuznetsov D. (2017). Rapid Biosynthesis of AgNPs Using Soil Bacterium *Azotobacter vinelandii* With Promising Antioxidant and Antibacterial Activities for Biomedical Applications. JOM.

[26] Jemal K., Sandeep B.V., Pola S. (2017). Synthesis, characterization, and evaluation of the antibacterial activity of *Allophylus serratus* leaf and leaf derived callus extracts mediated silver nanoparticles. J. Nanomater..

[27] Dehaj M.S., Mohiabadi M.Z. (2019). Experimental study of water-based CuO nanofluid flow in heat pipe solar collector. J. Therm. Anal. Calorim..

[28] Karthik L., Kumar G., Kirthi A.V., Rahuman A.A., Rao K.V.B. (2014). *Streptomyces* sp. LK3 mediated synthesis of silver nanoparticles and its biomedical application. Bioprocess Biosyst. Eng..

[29] Govarthanan M., Selvankumar T., Manoharan K., Rathika R., Shanthi K., Lee K.J., Cho M., Kamala-Kannan S., Oh B.T. (2014). Biosynthesis and characterization of silver nanoparticles using panchakavya, an Indian traditional farming formulating agent. Int. J. Nanomed..

[30] Lanje A.S., Sharma S.J., Pode R.B., Ningthoujam R.S. (2010). Synthesis and optical characterization of copper oxide nanoparticles. Adv. Appl. Sci. Res..

[31] Erjaee H., Rajaian H., Nazifi S. (2017). Synthesis and characterization of novel silver nanoparticles using *Chamaemelum nobile* extract for antibacterial application. Adv. Nat. Sci. Nanosci. Nanotechnol..

[32] Gouyau J., Duval R.E., Boudier A., Lamouroux E. (2021). Investigation of Nanoparticle Metallic Core Antibacterial Activity: Gold and Silver Nanoparticles against *Escherichia coli* and *Staphylococcus aureus*. Int. J. Mol. Sci..

[33] John M.S., Nagoth J.A., Zannotti M., Giovannetti R., Mancini A., Ramasamy K.P., Miceli C., Pucciarelli S. (2021). Biogenic Synthesis of Copper Nanoparticles Using Bacterial Strains Isolated from an Antarctic Consortium Associated to a Psychrophilic Marine Ciliate: Characterization and Potential Application as Antimicrobial Agents. Mar. Drugs.

[34] Yin I.X., Zhang J., Zhao I.S., Mei M.L., Li Q., Chu C.H. (2020). The Antibacterial Mechanism of Silver Nanoparticles and Its Application in Dentistry. Int. J. Nanomed..

[35] Meghana S., Kabra P., Chakraborty S., Padmavathy N. (2015). Understanding the pathway of antibacterial activity of copper oxide nanoparticles. RSC Adv..

[36] Chen M.C., Koh P.W., Ponnusamy V.K., Lee S.L. (2022). Titanium dioxide and other nanomaterials based antimicrobial additives in functional paints and coatings: Review. Prog. Org. Coatings.

[37] Solano R., Patiño-Ruiz D., Herrera A. (2020). Preparation of modified paints with nano-structured additives and its potential applications. Nanomater. Nanotechnol..

[38] Hajipour P., Eslami A., Bahrami A., Hosseini-Abari A., Saber F.Y., Mohammadi R., Mehr M.Y. (2021). Surface modification of TiO_2_ nanoparticles with CuO for visible-light antibacterial applications and photocatalytic degradation of antibiotics. Ceram. Int..

[39] Lu J., Fan X., Hu J., Li J., Rong J., Wang W., Chen Y., Liu W., Chen J., Chen Y. (2023). Construction and function of robust and moist bilayer chitosan-based hydrogel wound dressing. Mater. Des..

[40] Lu J., Chen Y., Ding M., Fan X., Hu J., Chen Y., Li J., Li Z., Liu W. (2022). A 4arm-PEG macromolecule crosslinked chitosan hydrogels as antibacterial wound dressing. Carbohydr. Polym..

[41] Li Y., Xia X., Hou W., Lv H., Liu J., Li X. (2023). How Effective are Metal Nanotherapeutic Platforms Against Bacterial Infections? A Comprehensive Review of Literature. Int. J. Nanomed..

[42] Vaja (Dumitru) F., Comanescu C., Oprea O., Ficai D., Guran C. (2012). Effects of ZnO nanoparticles on the wet scrub resistance and photocatalytic properties of acrylic coatings. Rev. Chim..

[43] Ficai D., Oprea O., Ficai A., Holban A. (2014). Metal Oxide Nanoparticles: Potential Uses in Biomedical Applications. Curr. Proteom..

